# Targeting CK2 eliminates senescent cells and prolongs lifespan in *Zmpste24*-deficient mice

**DOI:** 10.1038/s41419-024-06760-0

**Published:** 2024-05-30

**Authors:** Jie Zhang, Pengfei Sun, Zhuping Wu, Jie Wu, Jiali Jia, Haoman Zou, Yanzhen Mo, Zhongjun Zhou, Baohua Liu, Ying Ao, Zimei Wang

**Affiliations:** 1 Guangdong Key Laboratory of Genome Stability and Human Disease Prevention, Carson International Cancer Center, Department of Biochemistry & Molecular Biology, School of Basic Medical Sciences, Shenzhen University Medical School, Shenzhen, 518055 China; 2 Guangdong Key Laboratory for Biomedical Measurements and Ultrasound Imaging, National—Regional Key Technology Engineering Laboratory for Medical Ultrasound, School of Biomedical Engineering, Shenzhen University Medical School, Shenzhen, 518060 China; 3 Shenzhen Key Laboratory for Systemic Aging and Intervention, National Engineering Research Center for Biotechnology (Shenzhen), Shenzhen University, Shenzhen, 518055 China; 4 School of Biomedical Sciences, Li Ka Shing Faculty of Medicine, The University of Hong Kong, 21 Sassoon Road, Pokfulam, Hong Kong

**Keywords:** DNA damage response, Stress signalling

## Abstract

Senescent cell clearance is emerging as a promising strategy for treating age-related diseases. Senolytics are small molecules that promote the clearance of senescent cells; however, senolytics are uncommon and their underlying mechanisms remain largely unknown. Here, we investigated whether genomic instability is a potential target for senolytic. We screened small-molecule kinase inhibitors involved in the DNA damage response (DDR) in *Zmpste24*^−/−^ mouse embryonic fibroblasts, a progeroid model characterized with impaired DDR and DNA repair. 4,5,6,7-tetrabromo-2-azabenzamidazole (TBB), which specifically inhibits casein kinase 2 (CK2), was selected and discovered to preferentially trigger apoptosis in *Zmpste24*^−/−^ cells. Mechanistically, inhibition of CK2 abolished the phosphorylation of heterochromatin protein 1α (HP1α), which retarded the dynamic HP1α dissociation from repressive histone mark H3K9me3 and its relocalization with γH2AX to DNA damage sites, suggesting that disrupting heterochromatin remodeling in the initiation of DDR accelerates apoptosis in senescent cells. Furthermore, feeding *Zmpste24*-deficient mice with TBB alleviated progeroid features and extended their lifespan. Our study identified TBB as a new class senolytic compound that can reduce age-related symptoms and prolong lifespan in progeroid mice.

## Introduction

Aging is a major risk factor for many chronic diseases, such as cardiovascular diseases, degenerative diseases, and cancer. These chronic diseases seldom exist discretely in older people, which makes it challenging for clinical strategies to treat specific diseases and resolve various symptoms resulting from comorbid conditions. Interventions that impede the aging process itself offer an alternative approach that may delay the onset of age-related chronic diseases and thus prolong healthy longevity. Cellular senescence is a process that helps to prevent damaged cells from proliferating, thereby protecting cells from transformation and maintaining tissue homeostasis [1]. Senescence is also an important trigger of tissue remodeling during embryonic development and in response to tissue damage; senescence-related factors recruit immune cells to promote tissue regeneration [2]. However, the rate of senescent cell accumulation increases with age, resulting in pervasive damage and deficient cell clearance that disturbs tissue homeostasis. An increased burden of senescent cells in various tissues is a major factor contributing to aging and age-related diseases [3, 4]. Removing senescent cells as a therapeutic strategy was first verified by killing cells with high *p16*^*Ink4a*^ expression in INK-ATTAC and p16-3MR transgenic mice, which delayed organ dysfunction and prolonged the lifespan in mice aged both prematurely and naturally [5]. Although this opened a new approach to anti-aging, only ‘late senescence’ cells that express a high level of *p16*^*Ink4a*^ can be killed limiting the feasibility and potential application of this approach in humans.

A pharmacological target removal approach has subsequently become the new focus. Small-molecule compounds, termed senolytics, which increase the death rate of senescent cells, can alleviate inflammation and age-related organ dysfunction by targeting diverse survival pathways. Tens of senolytics have been reported. For instance, dasatinib, either alone or combined with quercetin, inhibits tyrosine kinase activity and initiates death in certain senescent cell types [6, 7]. Fisetin and quercetin are natural flavonoids that affect the PI3K/AKT/mTOR pathway; navitoclax (ABT-263) and ABT-737 inhibit the anti-apoptotic protein family Bcl2/BCL-xL, consequently triggering mitochondrial-mediated apoptosis [8, 9], and the cardiac glycosides ouabain has a similar effect. In addition, FOXO4-related peptide inhibits the interaction of p53 with FOXO4 to promote p53-medicated apoptosis [10], and the HSP90 inhibitor 17-DMAG impacts the anti-apoptotic PI3K/AKT pathway [11]. Galactose-modified cytotoxic prodrugs preferentially target senescent cells because these have characteristically high expression of β-galactosidase. Interestingly, panobinostat, a histone deacetylase inhibitor, has been found to promote selective apoptosis of senescent cells by increasing acetylation of histone H3 and reduced the expression of Bcl-xL [12]. Although several of the most promising senolytics are already being evaluated in preclinical studies, senolytics are still relatively uncommon and their underlying mechanisms remain largely unknown – new classes need to be identified.

The strength and extent of DNA damage determines the cell fate, that is, activation of DNA repair and subsequent cell survival or elimination of the severely damaged cells through programmed cell death. Cells confronted with DNA damage have different options to react to this hazard. Possible cellular responses are frequently mutually exclusive ones [13, 14]. Genomic instability is one of the four causal hallmarks of aging [15]. Chemotherapeutic agents, ionizing radiation, metabolites, and replication stresses cause continual DNA damage, which if repaired incorrectly may lead to somatic mutations and thus cell transformation or, if accumulated, can result in constant activation of the DNA damage response (DDR) and cause senescence. Pertinently, premature aging syndromes such as Hutchinson-Gilford progeria syndrome (HGPS), Werner syndrome, Bloom syndrome, Cockayne syndrome and ataxia telangiectasia are characterized by genomic instability. Ourselves and others have shown that an accumulation of DNA damage owing to impaired DDR and DNA repair is a hallmark feature of HGPS and *Zmpste24*-deficient mice [16–18]. On the other hand, massive DNA damage, for example in chemotherapy and radiotherapy, may directly lead to apoptosis [19]. Congruently, severely defective DDR and/or DNA repair causes embryonic or perinatal lethality, as seen in *Rad51* deficient mice [20]. Although the precise molecular mechanisms that prompt cells to become senescent or apoptotic remain unclear, inducing the senescence-to-apoptosis transition could be an important way to eliminate senescent cells. To investigate this hypothesis, we screened small-molecule compounds library that target DDR-related kinases in *Zmpste24*^−/−^ mouse embryonic fibroblasts (MEFs). Our research identified 4,5,6,7-tetrabromo-2-azabenzamidazole (TBB), a casein kinase 2 (CK2) inhibitor, as a promising senolytic drug to kill DDR-deficient senescent cells and delay premature aging.

## Results

### Screening senolytic potential targeting DDR-related kinases

**Fig. 1:**
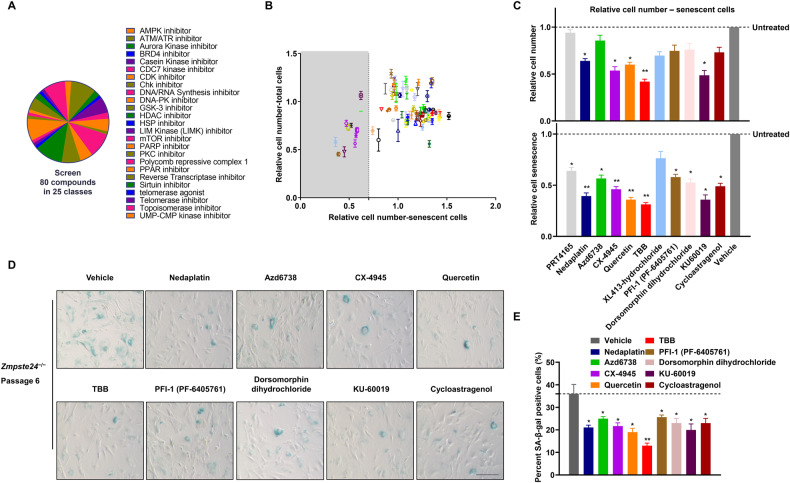
Screening anti-aging targets of DDR-related kinase inhibitors in *Zmpste24*-deficient MEFs. **A** A Pie chart indicating the different functional groups of drugs in the DDR-related kinase inhibitors library used in the screen. **B** The primary screen of all 80 DDR-related kinase inhibitors at 10 μM concentration. X-axis – number of senescent cells in the drug-treated cultures relative to cells treated with vehicle only; Y-axis – fraction of total cells remaining after drug treatment relative to vehicle-treated controls; gray shaded area shows drugs that reduce the proportion of senescent cells by > 70%. **C** Independent validation of the primary screen expressed as cell senescence and cell number relative to untreated control cultures of senescent cells. All drugs were used at 10 μM, chart plots mean ± SD from triplicate experiments. *p < 0.05, **p < 0.01 by two-tailed Student’s *t* test. **D** Representative images derived from triplicate experiments of passage 6 *Zmpste24*^−/−^ MEF cultures measuring senescence-associated β-galactosidase (SA-β-Gal) activity using X-gal staining. Scale bar, 50 μm. **E** Quantification of SA-β-Gal–positive staining from 10 randomly chosen fields of view for each group. The chart plots mean ± SEM. *p < 0.05, **p < 0.01.

Understanding the fate of cells in the case of DNA damage, whether to maintain cell proliferation or to senescence and apoptosis, is of great significance for anti-aging and anti-tumor research. Recent studies have revealed that kinases can act as molecular effectors that direct response pathways toward senescence or apoptosis [21]. To identify compounds exerting senolytic effects via the DDR-related cell fate pathway, we screened 80 molecules with defined inhibitory effects on DDR-related kinases. This chemical library covered 25 pathways in 7 different functional classes in *Zmpste24*^−/−^ MEFs – a classic progeroid cell model that shows early cell senescence in vitro [22–24] (Fig. 1A and Supplementary Fig. S1A). The primary screen was performed at 10 μM concentration for each compound by analyzing fluorescence staining to quantify senescence-associated β-galactosidase (SA-β-Gal) activity (Supplementary Fig. S1B). Two days after drug treatment, the number of senescent cells was plotted against the total cell number. While most compounds increased the senescent cell count, indicating possible senescence promoting effects, only 11 compounds significantly reduced the senescent cell fraction to below 70%, suggesting therapeutic senescence clearing potential (Fig. 1B). These 11 drugs were retested in triplicate at 10 μM to validate the results of the primary screen. However, 5 of these 11 drugs did not significantly alter the total cell number, which might suppress senescence phenotypes, whereas 5 significantly reduced both senescent cell and total cell numbers (Fig. 1C), and were consequently considered to have senolytic potential. It was also found that one of these drugs, XL413-hydrochloride, had no apparent effect on senescence phenotypes. Next, SA-β-Gal fluorescence staining assays were performed to appraise the effect of drug treatment on cell viability (Fig. 1D). The most significant reductions in the ratio of senescent to non-senescent cells was observed for quercetin, Ku-60019, CX-4945 and TBB (4,5,6,7-tetrabromo-2-azabenzamidazole) (Fig. 1E and Supplementary Fig. S1C). Notably, quercetin is a typical senolytic and extends healthy life in vivo [25, 26]. Ku-60019 is an inhibitor of ATM, a master regulator of DDR [27]. CX-4945 and TBB inhibit CK2 [28], which phosphorylates a variety of proteins that regulate DDR, circadian rhythms, cell survival, senescence, and apoptosis. TBB treatment reduced SA-β-Gal staining by more than 50%, which was more effective than quercetin; hence we selected TBB as a promising senolytic potential for further study.

### TBB induces apoptosis of senescent cells in *Zmpste24*-deficient MEFs

**Fig. 2:**
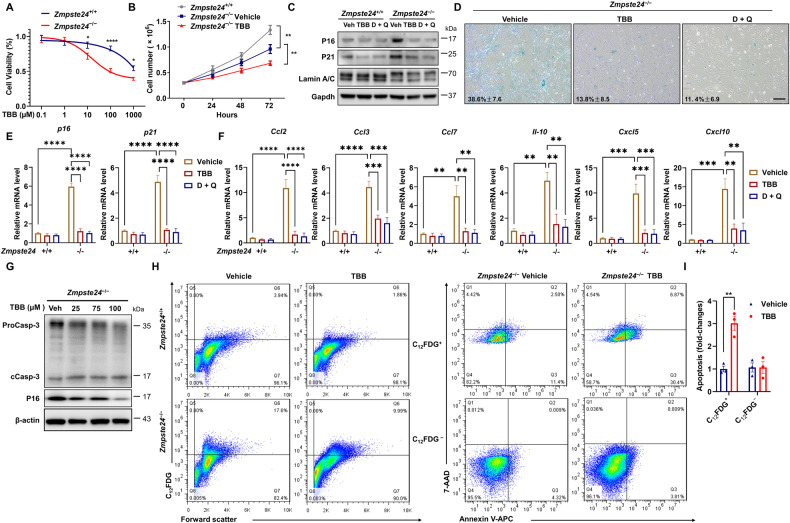
TBB ameliorates the senescence phenotype by accelerating the apoptosis of senescent cells in *Zmpste24*-deficient MEFs. **A** MEF cells were treated with the indicated amounts of TBB for 24 h. Cell viability was assessed with an MTS assay. **B** Cell proliferation was measured in *Zmpste24*^−/−^ MEFs and wild-type littermate controls treated with 75 μM TBB or DMSO (control). **C** Representative western blot analysis of P16, and P21 in *Zmpste24*-deficient cells in *Zmpste24*^–/–^ MEFs and wild-type littermate controls after treatment with vehicle (DMSO), TBB (75 μmol/L) and D + Q (D: 200 nmol/L; Q: 20 μmol/L) at the indicated concentration. **D** Representative images of SA-β-Gal activity showing blue-stained senescent cells in *Zmpste24*^−/−^ MEFs treated with 75 μM TBB, D + Q (D: 200 nmol/L; Q: 20 μmol/L) or DMSO (control) at passage 6. Scale bar, 100 μm. **E** Quantitative RT-PCR analysis of *p16* and *p21* mRNA levels in *Zmpste24*^*−/−*^ and wild-type littermate control after treatment with vehicle (DMSO), TBB (75 μmol/L) and D + Q (D: 200 nmol/L; Q: 20 μmol/L) at the indicated concentration. The data represent the means ± SEM. **F** Quantitative RT-PCR analysis of SASP genes levels in *Zmpste24*^−/−^ and wild-type littermate control after treatment with vehicle (DMSO), TBB (75 μmol/L) and D + Q (D: 200 nmol/L; Q: 20 μmol/L) at the indicated concentration. The data represent the means ± SEM. **G** Representative western blot analysis of proCaspase-3 (ProCasp-3), cleaved caspase-3 (cCasp-3), P16 and β-actin in *Zmpste24*-deficient cells after treatment with vehicle or TBB at the indicated concentration. **H** Flow cytometric analysis by Annexin V/7-AAD staining of cell death of senescent *Zmpste24*^−/−^ MEF cells cultures treated with TBB; Histogram of senescent *Zmpste24*^−/−^ MEF cells and wild-type littermate controls treated with 75 μM TBB or vehicle only (left). And right is *Zmpste24*^−/−^ MEF cells are either senescent (C_12_FDG^+^; top) or non-senescent (C_12_FDG^−^, bottom). Live cells were double negative for 7-AAD and Annexin V (bottom left quadrant), early apoptotic cells were positive for Annexin V (bottom right quadrant), late apoptotic cells were positive for Annexin V and 7-AAD (top left quadrant) and dead cells were positive for 7-AAD only (top right quadrant). **I** Quantification of flow cytometry data. Apoptosis of senescent and non-senescent cells was calculated by summing up all Annexin V-positive cells. Chart plots mean ± SEM from triplicate experiments, **p < 0.01, two-tailed Student’s *t* -test.

To affirm the specificity of TBB on senescent versus non-senescent cells, wild-type (WT) and *Zmpste24*-deficient MEFs (passage 6) (Supplementary Fig. S2A) were treated with 0−1000 μM TBB for 24 h. The potting concentration-effect curve by MTS assay showed that TBB inhibited cell viability in a dose-dependent manner (Fig. 2A). The IC_50_ of approximately 1000 μmol/L for WT MEFs was significantly reduced to 100 μmol/L in *Zmpste24*-deficient MEFs (Fig. 2A), implying that TBB is more effective in inducing cell death in senescent cells. Though cell growth retardation was found in 75 μmol/L TBB-treated *Zmpste24*^−/−^ MEFs (Fig. 2B), the cells exhibited a much younger phenotype compared with vehicle control (Fig. 2C, D), since the proportion of SA-β-Gal positive cells decreased and the expression of senescence marker p16 and p21 decreased (dasatinib (D) + quercetin (Q) is a senolytic positive control). Meanwhile, no significant changes were observed in the percentage of SA-β-Gal positive cells in WT cells (Supplementary Fig. S2B). This result attracted our attention, leading us to investigate whether severe inhibition of CK2 activity could readily induce the senescent cell apoptosis, similar to the effect of senolytic. We simultaneously monitored approximately 20 genes associated with the SASP, as well as age-related marker genes p16 and p21. The results showed that severe inhibition of CK2 activity could reduce the expression of some SASP genes in cultured *Zmpste24*- deficient cells, as shown in Fig. 2E, F. We reasoned that TBB might preferentially trigger apoptosis of senescent cells and investigated this hypothesis by using different concentrations of TBB to treat *Zmpste24*-deficient MEFs and quantifying their apoptosis. We observed a dose-dependent increase in the level of cleaved caspase 3 and a decrease in the level of senescence marker p16 (Fig. 2G), indicating that TBB induced apoptosis in progeroid cells is correlated with ameliorating the senescent phenotype. To determine whether TBB inhibition preferentially triggers apoptosis of senescent cells, *Zmpste24*^−/−^ MEFs treated with TBB were stained for C_12_FDG to label senescent cells, then stained for Annexin V, an early apoptosis marker, and 7-aminoactinomycin D (7-AAD), a membrane impermeable dye that is generally excluded from viable cells as a later apoptosis marker, and finally analyzed with flow cytometry. The gating strategy for C_12_FDG-Annexin V-7AAD flow cytometry was shown in Supplementary Fig. S2C. After TBB treatment, the proportion of C_12_FDG positive cells decreased from 17.6% to 9.99% (Fig. 2H, left), while the proportion of Annexin V and 7-AAD positive cells increased from 13.79% to 37.03% in *Zmpste24*^−/−^ MEFs (Fig. 2H, right, top), Moreover, the apoptotic proportion of C_12_FDG negative cells did not change significantly in *Zmpste24*^−/−^ MEFs (Fig. 2H, right, bottom). In contrast, there was no significant increase in apoptosis of C_12_FDG-negative cells, supporting the conclusion that TBB selectively triggers apoptosis in senescent cells (Fig. 2I).

### TBB aggravates the accumulation of DNA damage in *Zmpste24*-deficient cells

**Fig. 3:**
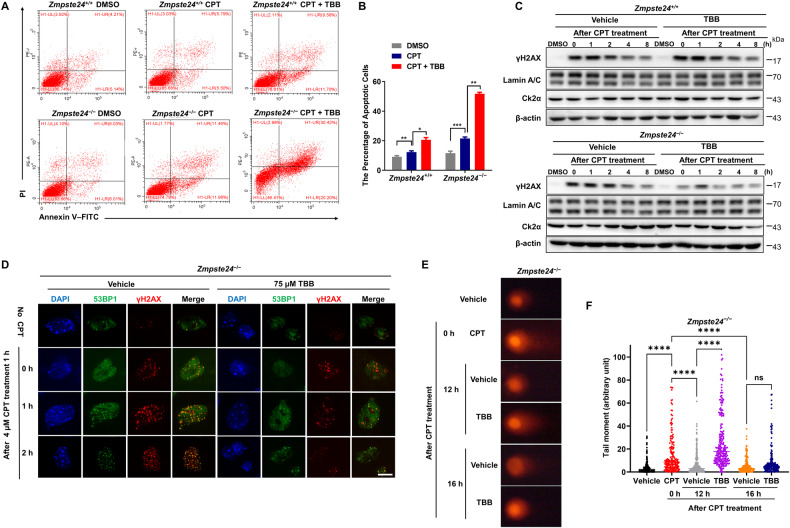
TBB induces apoptosis in *Zmpste24*-deficient cells by aggravating the accumulation of DNA damage. **A** Representative flow cytometric plots to measure apoptosis: viable (gate II: PI^−^ annexin V^−^) and apoptotic (gates III and IV: PI^−^ annexin V^+^ and PI^+^ annexin V^+^) (right) cells of *Zmpste24*^−/−^ MEFs and wild-type littermate controls treated with 4 μM camptothecin (CPT) for 1 h to induce DNA damage or vehicle DMSO control pretreated with 75 μM TBB or mock DMSO (control) for 6 h at passage 6. **B** Quantification of the percentage of apoptotic cells after treatment with vehicle or TBB, as in **A**. *p < 0.05, **p < 0.01, ***p < 0.001. **C** Representative immunoblots from at least three independent experiments showing protein levels of γH2AX at indicated time points after 4 μM CPT for 1 h induced DNA damage or vehicle DMSO control in *Zmpste24*^−/−^ MEFs and wild-type littermate controls treated with 75 μM TBB for 6 h. **D** Representative immunofluorescence confocal microscopy images of γ-H2AX and 53BP1 immunofoci recruitment for each condition in response to 4 μM CPT-induced DNA damage in TBB treated *Zmpste24*^−/−^ MEFs. TBB delayed recruitment of 53BP1 to γ-H2AX at the indicated time (after CPT treatment 0, 1, 2 h). Scale bar, 10 µm. **E** Representative comet assay images at indicated time points after 4 μM CPT for 1 h to induce DNA damage or vehicle DMSO control in *Zmpste24*^−/−^ MEFs pretreated with 75 μM TBB for 6 h. **F** Comet tail moments quantified by Open Comet software after treatment with vehicle or TBB, as in **D**. Chart plots mean ± SEM, ****p < 0.0001, *n* = 150.

Defective DDR is an important feature of *Zmpste24*-deficent cells. To understand how TBB induces apoptosis in senescent cells, passage (P) 6 senescent *Zmpste24*^−/−^ MEFs were pretreated with TBB, and then temporarily treated for 1 h with camptothecin (CPT), a chemical reagent typically used to induce DNA breaks, followed by flow cytometry analysis after Annexin V staining. CPT treatment alone led to an increased proportion of Annexin V positive cells (23.4%) in *Zmpste24*^−/−^ MEFs, and when pretreated with TBB, the percentage of Annexin V positive cells was further increased to 50.6% (Fig. 3A, B, p < 0.001). These results indicate that TBB accelerates *Zmpste24*-deficient MEFs apoptosis in the presence of DNA damage. Western blot showed that TBB did not affect DDR marker γ-H2AX level in WT cells, by contrast, γ-H2AX level was significantly reduced in *Zmpste24*^−/−^ MEFs (Fig. 3C). Meanwhile, immunofluorescence staining results showed that the number of γ-H2AX foci that colocalized with 53BP1 foci was significantly less in the presence of TBB in *Zmpste24*^−/−^ MEFs (Fig. 3D). To ascertain whether the decrease of γ-H2AX was due to less DNA damage or impaired γ-H2AX response in the presence of TBB, a comet assay was performed to monitor the severity of DNA damage. Comet tail moments were significantly increased in *Zmpste24*^−/−^ MEFs upon CPT, which was even more pronounced in the presence of TBB (Fig. 3E, F), while the tail moment was not apparently different in wild-type cells versus vehicle (Supplementary Fig. S3A, B). Collectively, these results imply that TBB might impair the DDR, thereby killing senescent cells.

### CK2 inhibition impairs DDR signaling and induces apoptosis

**Fig. 4:**
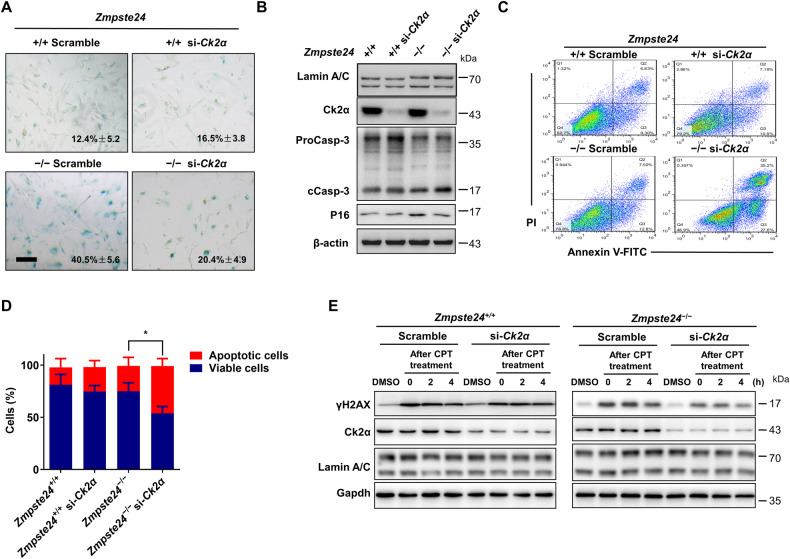
TBB-induced apoptosis in *Zmpste24*-deficient cells via inhibition of CK2. **A** Senescence-associated β-galactosidase staining in Ck2α or scramble siRNA treated *Zmpste24*^+/+^ and *Zmpste24*^−/−^ MEFs at passage 6 (scale bar, 200 μm). **B** Representative immunoblots showing the levels of proCaspase-3 (ProCasp-3), cleaved caspase-3 (cCasp-3) and P16 in Ck2α or scramble siRNA-treated *Zmpste24*^−/−^ MEFs and wild-type controls. **C** Representative flow cytometric plots for assessing apoptosis in Ck2α or scramble siRNA-treated *Zmpste24*^−/−^ MEFs and wild-type controls. **D** Quantitation of the percentage of viable (gate II: PI^−^/annexin V^−^) and apoptotic (gates III and IV: PI^−^/annexin V^+^ and PI^+^/annexin V^+^) cells in (**C**); chart plots mean ± SD from independent triplicate experiments. **E** Representative immunoblots showing protein levels of γH2AX at indicated time points after 4 μM camptothecin (CPT) for 1 h to induce DNA damage or vehicle DMSO control in siRNA-Ck2α transfected in *Zmpste24*^−/−^ MEFs and wild-type controls treated with 75 μM TBB for 6 h at the indicated time (after CPT treatment 0, 2, 4 h). At least three independent experiments were performed.

TBB is widely used as a specific inhibitor of CK2 [29]. Moreover, TBB can significantly inhibit the activity of CK2 in vitro (Supplementary Fig. S4A). Next, we determined whether TBB induces apoptosis of senescent cells by CK2α. CK2α siRNA-transfected WT and *Zmpste24*^−/−^ MEFs were prepared (Supplementary Fig. S4B). SA-β-gal staining showed that CK2α knockdown decreased the proportion of senescent cells from 40.5% in scrambled MEFs to 20.4% in CK2α siRNA-treated *Zmpste24*^−/−^ MEFs (Fig. 4A). Knockdown of CK2α increased the level of cleaved caspase 3 and decreased the p16 level in *Zmpste24*-deficient cells (Fig. 4B). By flow cytometric analysis, knocking down CK2α significantly induced apoptosis in *Zmpste24*^−/−^ MEFs, with the proportion of apoptotic cells increasing from 20.32% to 52.8% (Fig. 4C, D). Similar to TBB treatment, si-CK2α-transfected senescent cells exhibited reduced expression of γ-H2AX in response to DNA damage (Fig. 4E). These findings suggest that CK2 inhibition impairs the DDR and induces apoptosis in *Zmpste24*-deficient senescent MEF cells.

### CK2 phosphorylates HP1α to promote chromatin remodeling upon DNA damage

**Fig. 5:**
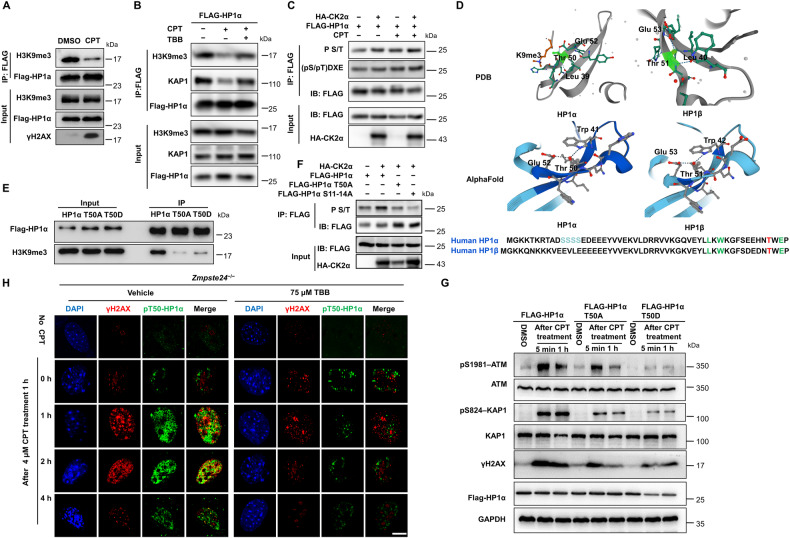
TBB affects the phosphorylation of HP1α T50 by CK2α, leading to an impaired DNA damage response in *Zmpste24*-deficient MEFs. **A** Immunoblots showing H3K9me3 protein in anti-Flag immunoprecipitates in HEK293 cells expressing FLAG-HP1α treated with 4 μM CPT for 1 h. **B** Representative immunoblots from at least three independent experiments showing HEK293 cells expressing FLAG-HP1α treated with 4 μM camptothecin (CPT) for 1 h to induce DNA damage or vehicle DMSO control pretreated with 75 μM TBB or mock DMSO (control) for 6 h. **C** Immunoblots showing p-S/T or phospho-CK2 Substrate [(pS/pT)DXE] of FLAG-HP1α transfected HA-CK2α and FLAG-HP1α treated with 4 μM camptothecin (CPT) for 1 h to induce DNA damage or vehicle DMSO control by using an anti-FLAG antibody immunoprecipitates in HKE293 cells. **D** A model for the interaction of the human HP1α (left) and HP1-β (right) chromodomain bound to methylated K9 of histone H3 (K9me3), based on the PDB protein database and AlphaFold. A network of hydrogen bonds between the side chains of Glu 52, Thr 50 and Leu 39 of the human HP1-α chromodomain; Glu 53, Thr 51 and Leu40 of the human HP1-β chromodomain. **E** Immunoprecipitation showing the binding capacity between H3K9me3 and various FLAG-HP1α mutants. **F** Immunoblots showing p-S/T of FLAG-HP1α or FLAG-HP1α mutants transfected HA-CK2α by using an anti-FLAG antibody in HKE293 cells. **G** Representative immunoblots showing protein levels of ATM-Ser1981, KAP1-Ser824 and γH2AX in FLAG-HP1α and T50A or T50D mutants transfected in HEK293 cells. Note that the response to DNA repair is less sensitive in FLAG-HP1α T50A or T50D mutants because of lower ATM-Ser1981, KAP1-Ser824 and γH2AX expression. Cells were treated with 4 μM camptothecin (CPT) for 1 h or vehicle DMSO and then analyzed by western blotting. Data represent independent triplicate experiments. **H** Representative immunofluorescence confocal microscopy images of γ-H2AX and pT50-HP1α immunofoci recruitment for each condition in response to 4 μM CPT-induced DNA damage in TBB treated *Zmpste24*^–/–^ MEFs. TBB inhibited the co-immunofoci of pT50-HP1α and γ-H2AX at the indicated time (after CPT treatment 0, 1, 2, 4 h). Scale bar, 10 µm.

Impaired heterochromatin remodeling complex HP1α/KAP-1 partially, if not entirely, underlies the defective DDR and accelerated senescence in *Zmpste24*-deficient cells [30–32]. HP1α phosphorylation promotes its nucleosome binding and γH2AX formation at DNA damage sites [33, 34]. We detected the binding ability between HP1α and H3K9me3, which interaction maintains heterochromatin structure and mediates chromatin remodeling in the DDR process. Co-immunoprecipitation experiments in HEK293 cells overexpressing HP1α-FLAG confirmed the interaction of HP1α and H3K9me3, but their interaction was obviously weakened upon CPT treatment (Fig. 5A). When treated with TBB, this process can be significantly inhibited (Fig. 5B). As CK2α has the potential to phosphorylate HP1α, we investigated the precise effect of CK2α on HP1α by over-expressing HP1α-FLAG with or without CK2α-HA in HEK293 cells. Anti-FLAG immunoprecipitation and western blotting revealed an obvious increase of phosphorylated S/T (p-S/T) level in a CK2α-HA dose-dependent manner (Supplementary Fig. S5A, B). Moreover, we found that CK2α can enhance the phosphorylation of HP1α upon CPT treatment (Fig. 5C). Studies have reported that phosphorylation of the HP1β T51 site by CK2 is particularly important for regulating the DNA damage response and repair of heterochromatin, and abnormal phosphorylation of HP1β Thr 51 site disrupts the interaction between HP1 and H3K9me3 [35]. Sequence alignment analysis revealed a CK2 phosphorylation motif centered on the T50 residue, which is highly conserved across species (Supplementary Fig. S5C). We used the Protein Data Bank (PDB) database and AlphaFold to compare and analyze the structure of HP1α and HP1β, and the results showed that HP1α Thr 50 site and HP1β Thr 51 site were highly similar in spatial structure (Fig. 5D). Notably, both inactivated (T50A) and constitutively activated (T50D) HP1α inhibited the interaction between HP1α and H3K9me3 (Fig. 5E). Studies have shown that the N-terminal of HP1α is also phosphorylated by CK2α, which promotes its binding to H3K9me3 [36]. Our results showed that the mutation of S11-14 to alanine (S11-14A) or T50 to alanine (T50A) sites both affected the phosphorylation level of HP1α by CK2α (Fig. 5F). Moreover, mutation of T50 to alanine (T50A) diminished the interaction of HP1α and CK2α (Supplementary Fig. S5D). This result suggested that HP1α T50 phosphorylation is critical for HP1α-H3K9me3 dissociation during the DDR. Both T50A and T50D mutants caused downregulation of pS1981-ATM and pS824-KAP-1 (Fig. 5G), which are key regulators of heterochromatin remodeling in DDR, further suggesting that CK2 mediated HP1α phosphorylation is important for initiating the DDR. Next, we examined pT50-HP1α and γH2AX foci after TBB treatment in *Zmpste24*^−/−^ MEFs. Remarkably, Immunofluorescence staining showed that TBB treatment inhibited pT50-HP1α and γH2AX foci formation and their colocalization (Fig. 5H), leading to impaired γH2AX foci response at sites of DNA damage in *Zmpste24*^−/−^ MEFs, but merely affected their foci formation and colocalization in WT cells (Supplementary Fig. S5E). Collectively, these findings suggest that CK2α phosphorylates HP1α, which promotes its dynamic dissociation from H3K9me3 and relocalization with γH2AX to DNA damage sites following DDR signaling transduction; TBB inhibits CK2α thus causing DNA damage to accumulate in *Zmpste24*^−/−^ MEFs.

### TBB treatment ameliorates progeroid features and extends lifespan in *Zmpste24*-deficient mice

**Fig. 6:**
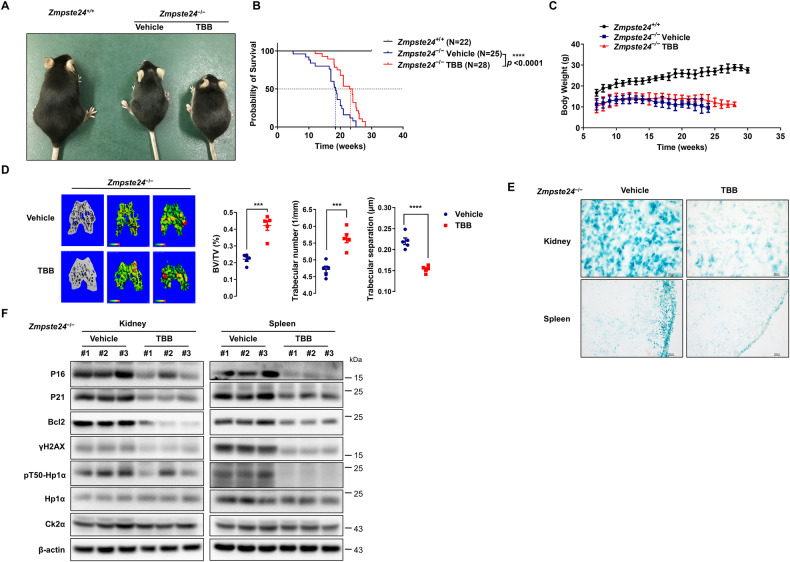
TBB ameliorates progeroid features and extends lifespan of *Zmpste24*-deficient mice. **A** *Zmpste24*^+/+^, *Zmpste24*^−/−^, and TBB-fed *Zmpste24*^−/−^ mice at 4 months old; TBB (50 μM/L) or vehicle DMSO was dissolved in drinking water daily. **B** Survival and life span of TBB-fed *Zmpste24*^−/−^ mice (*n* = 28) compared with those of the vehicle-fed controls (*n* = 25) using Kaplan–Meier analysis. A survival rate of 50% was observed at 18 and 24 weeks respectively in *Zmpste24*^−/−^ and TBB-fed *Zmpste24*^−/−^ mice. **C** Body weight curves of male, TBB-fed *Zmpste24*^−/−^ mice (*n* = 11) were compared with vehicle controls (*n* = 9) or wild-type mice (*n* = 5). **D** Micro-CT analysis showing increased trabecular bone volume/tissue volume, trabecular number, and reduced trabecular separation in TBB-fed *Zmpste24*^−/−^ mice (*n* = 5) compared with those of the vehicle-fed controls (*n* = 5). ***p < 0.001, ****p < 0.0001, two-tailed Student’s *t* -test. **E** Representative photos of SA-β-gal activity showing blue-stained senescent cells of kidney and spleen in TBB-fed *Zmpste24*^−/−^ mice (*n* = 5) compared against those of vehicle-fed controls (*n* = 5). Scale bar, 50 μm. **F** Immunoblots showing P16, P21 and Bcl2 protein levels in different tissues isolated from male TBB-fed *Zmpste24*^−/−^ and vehicle-fed control mice.

To test whether TBB can attenuate premature aging phenotypes in mice, 50 μmol/L TBB was added to drinking water fed to *Zmpste24*^−/−^ mice and their WT littermates. The treatment started at age 1 month and continued through the whole lifetime; the body weight of the mice and their survival lifespan were regularly monitored. The survival curve comparison of the untreated and TBB-fed mice by Kaplan–Meir analysis revealed that the median lifespan increased from 18 (untreated mice) to 24 weeks in TBB-treated mice (Fig. 6A, B), while no difference in body weight was observed (Fig. 6C). The micro-computed tomography (CT) analysis identified an increased ratio of trabecular bone volume/tissue volume, increased trabecular number, and decreased trabecular separation in TBB-treated *Zmpste24*^−/−^ mice (Fig. 6D and Supplementary Fig. S6A). Notably, the number of SA-β-gal positive cells, and p16 and p21 levels were significantly reduced in the kidney and spleen compared with the vehicle control, while the anti-apoptosis protein Bcl-2 was decreased (Fig. 6E, F, Supplementary Fig. S6B, C). We used TUNEL staining to detect the effect of TBB on apoptosis in tissue in vivo, and the results showed that TBB could slightly induce apoptosis in vivo, compared with control mice (Supplementary Fig. S7). In summary, this research demonstrated that TBB alleviated progeroid features and extended the lifespan of *Zmpste24*-deficient mice by promoting apoptosis of senescent cells.

## Discussion

Accruing evidence suggests that senolytic clearance may be a promising therapeutic strategy to affect the aging process and potentially treat age-related pathologies. Here, we established an SA-β-gal colorimetric and florescent assay in *Zmpste24*-deficient MEFs as a screening platform to identify senolytic potentials. Considering that DDR and DNA repair deficiency might lead cells to follow senescence or apoptotic pathways, we screened a molecular library of DDR-related kinase inhibitors. Among 80 compounds, we identified CK2 inhibitors, exemplified by TBB, as a new class of senolytic potential. TBB preferentially induced apoptosis in senescent cells and alleviated progeroid features of *Zmpste24*-deficient mice and extended their lifespan. Mechanistically, CK2 phosphorylates heterochromatin HP1α, which promotes its dynamic dissociation with H3K9me3 and relocalization at DNA double-strand break sites (DSB), which facilitates γH2AX dispersion. CK2 inhibition by TBB aggravates extant DDR defects in senescent cells and results in apoptosis. TBB is a CK2 specific inhibitor with very lower affinity to other known proteins, that fails to induce apoptosis in cells expressing inhibitor-resistant CK2 [37]. Therefore, our findings support CK2 inhibitors as a new senolytic class and show the potential of TBB to treat premature aging syndromes such as HGPS.

Anti-apoptotic pathways of senescent cells, such as AKT/mTOR, Bcl2/BCL-xL, FOXO, and p53, are frequently investigated as senolytic targets. DNA damage is a major cause of cell senescence and apoptosis, though it remains unknown how the DDR pathway affects the disposition between senescent and apoptotic states. Exploring how apoptosis overcomes DNA damage induced senescence may provide a new way to identify senolytics. We applied this concept to identify 10 potential senotherapeutics, which mainly divided into two classes: senomorphics suppress senescence phenotypes, included Dorsomorphin dihydrochloride, PFI-1, PRT 4165, Azd6738 and cycloastragenol, all targeting the PI3K/AKT/mTOR pathway; and senolytics kill senescent cells, including TBB, CX-4945, quercetin, KU-60019 and Nedaplatin, which targeteted CK2, PI3K/AKT/mTOR, ATM/ATR and DNA synthesis pathways respectively. TBB and CX-4945 are specific CK2 inhibitors [28, 29]. Quercetin is a well-known flavonoid senolytic that targets the AKT/mTOR pathway as well as the CK2 pathway [38]. Pertinently, ATM is a master regulator of DDR that phosphorylates H2AX on Ser139 (γH2AX) to promote DDR signal transduction, and this process depends on CK2 [39–42]. Indeed, KU-10069, an ATM inhibitor, was previously screened as a senolytic candidate and reported to induce cancer cell apoptosis [43]. Both TBB and the classic senolytic quercetin have been shown to inhibit the activity of CK2 [44]. Meanwhile, dasatinib also can indirectly inhibit CK2 activity through SRC kinase [45]. It can be seen that TBB and classical senolytic D + Q can inhibit the activity of CK2. Our present data show that TBB itself did not induce DNA damage but rather affected the initiation of the DDR process, indicated by inhibited formation of γH2AX foci, which hindered further recruitment of DNA repair factors and thereby aggravated DNA damage and promoted apoptosis in senescent cells. Consequently, targeting the DDR pathway is a promising senolytic strategy. CK2 is a highly conserved, constitutively active serine/threonine protein kinase that sustains multiple survival-enhancing functions in cell cycle checkpoint, DDR, and apoptosis [46]. Upon ionizing radiation exposure, CK2 relocalizes from perinuclear structures to the nuclei, where it phosphorylates the mediator of DNA damage checkpoint protein 1 to effect transient chromatin anchorage of activated ATM together with the MRE11-RAD50-NBS1 complex, in turn enabling γH2AX formation and rapid dispersal to neighboring nucleosomes [47]. Higher order chromatin, for example, heterochromatin characterized by general hallmark HP1-H3K9me3 complex, impedes DDR signaling [48]. CK2 plays a vital role in regulating chromatin remodeling in the DDR process by serial dynamic post-translational modifications. We discovered that CK2 phosphorylates HP1α, allowing dynamic recruitment of HP1α to DNA damage breaks. The phosphorylation of HP1α induced by DNA damage is considered an important condition, which has been reported to coordinate most downstream chromatin-associated events, such as the formation of γH2AX foci [49]. Our present findings support this hypothesis and substantiate that CK2 directly regulates the heterochromatin HP1α-H3K9me3 interaction. Mutant HP1α T50 inhibits ATM-KAP1 phosphorylation and γH2AX formation, indicating that CK2-regulated pT50-HP1α coordinates ATM to promote heterochromatin γH2AX formation during DDR [50]. Intriguingly, TBB not only reduces the formation of pT50-HP1α/γH2AX foci, but also causes the mislocalization of pT50-HP1α and γH2AX following DDR inactivation, implying that inhibition of the CK2-HP1α axis accelerates serious DNA damage accumulation and obstacles overcoming from cell senescence to apoptosis. It is unclear why TBB selectively induces apoptosis in senescent (i.e., progeroid) cells. A plausible explanation would be that the dosage matters – a mild defect or CK2 inhibition causes senescence, while further deterioration or severe inhibition of CK2 leads to apoptosis. Congruently, our previous research showed that CK2 activity is downregulated in *Zmpste24*^−/−^ mice, and enhancing CK2 activity with spermidine alleviated premature aging features [51]. Now we have shown that feeding drinking water with 50 μM TBB also extends lifespan and ameliorates premature aging phenotypes in *Zmpste24*^−/−^ mice, accompanied with the downregulation in p16, p21, Bcl-2 and SA-β-gal activity in the kidney and spleen. Consistent with our data, another recent report showed that TBB supplementation extends the lifespan of *Caenorhabditis elegans* [52]. On the other front, CK2 inhibits apoptotic progression by phosphorylating caspase substrates to block their cleavage [53]. CK2 depletion reduces the formation of γH2AX foci in radiation therapy and causes cancer cell death [54]. It has also been shown that inhibition of CK2 activity improves IR-induced human mesenchymal stem cells (hMSC) senescence [55]. TBB and CX4945, as specific CK2 inhibitors, can induce apoptosis in many types of cancer cells and are promising chemotherapeutic agents for chemo-refractory tumors [56, 57]. Therefore, it is possible that ‘senolytic’ and ‘senomorphic’ effects may coexist, each with opposing activity functions on the same target, and that both ‘toxicity to senescent cells’ and ‘alleviation of cellular senescence’ could attenuate aging processes at the systemic level. Nonetheless, DNA damage severity and drug initiation timing must be carefully evaluated. CK2-based senolytic strategies merit further exploration and development especially regarding applications to healthy aging. To summarize, we have identified several DDR kinase inhibitors, exemplified by TBB, with evident senolytic potential to specifically induce apoptosis in prematurely senescent cells. TBB feeding alleviates premature aging phenotypes and extends lifespan in progeroid mice by inhibiting CK2α phosphorylation of HP1α in response to DNA damage. Together with our previous findings, this new evidence highlights CK2α as a dual target for ‘senolytic’ and ‘senomorphic’ strategies in anti-aging intervention and the senolytic potential of TBB to treat progeroid syndromes.

**Fig. 7:**
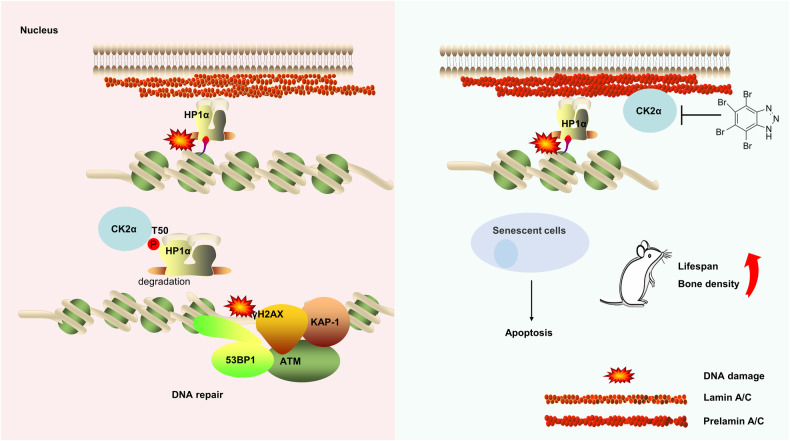
Working model. In normal cells undergoing DNA damage, CK2α regulates heterochromatin relaxation by phosphorylating HP1α, which promotes its dynamic dissociation with H3K9me3 and relocalization at DNA double-strand break sites (DSB), which facilitates DNA damage response and repair. Reducing CK2 activity leads to the accumulation of DNA damage, which leads to cellular senescence. Since CK2 activity is low in *Zmpste24*-deficient cells (progeria cells), CK2 inhibition by TBB aggravates extant DDR defects in senescent cells and results in apoptosis. CK2 activity determines the fate of cells undergoing varying degrees of DNA damage.

We propose a model (Fig. 7) that CK2α is particularly important for maintaining the DNA damage response and repair of heterochromatin. In normal cells, CK2α promotes the decondensed structure of heterochromatin by phosphorylating the HP1α in case of DNA damage, thus facilitating the DNA damage response and repair. However, due to the low activity of CK2 in progeria cells, treatment with CK2 inhibitor TBB (TBB itself does not act as an inducer of DNA damage) to further inhibit CK2 activity will lead to increased accumulation of DNA damage, thus leading to apoptosis of progeria cells, acting as a senolytics effect and delaying the phenotype of premature aging mice.

## Materials and methods

### Reagents and antibodies

Rabbit anti-CK2α (ab236664), anti-ATM (ab78), anti-γH2AX (ab81299), anti-Bcl2 (ab32124), anti-Kap-1 (ab10484), anti-p-KAP-1 (Ser824, ab70369), anti-phospho-(Ser/Thr) Phe (ab17464), anti-H3K9me3 (ab8898) were obtained from Abcam (Cambridge, UK). Anti-lamin A/C (sc-20681), anti-p21 (sc-6246), and anti-p16 (sc-1661) were purchased from Santa Cruz Biotechnology. Rabbit anti-γH2AX (05-636), anti-p-ATM (Ser1981) (05-740), anti-HP1α (05–689) were sourced from EMD Millipore. Mouse anti-p-ATM (Ser1981) (#5883), anti-cleaved caspase-3 (#9661) and Rabbit anti-phospho-CK2 Substrate [(pS/pT)DXE] (#8738) were purchased from Cell Signaling Technology (Beverly, MA). Mouse anti-HA, anti-Flag were obtained from Sigma-Aldrich. Alexa Fluor 488 goat anti-rabbit IgG (#A11008), Alexa Fluor 594 goat anti-mouse IgG (#A11005), ImaGene Green^TM^ C_12_FDG Kit (#I2904) were purchased from Thermo Fisher Scientific. Mouse anti-actin, anti-GAPDH were obtained from Beyotime. Anti-pT50-HP1α monoclonal antibodies were prepared by Abmart generating from a specific phosphorylated peptide (peptide sequence C-SEEHN(pT)WEPEK). The chemical library were purchased from Targetmol Chemicals Inc. Cellular Senescence Detection Kit was purchased from Cell Signaling Technology (#9860). FITC Annexin V Apoptosis Detection Kit I (#556547), and APC Annexin V Apoptosis Detection Kit I (#550475) were purchased from BD Pharmingen.

### Cell culture

The *Zmpste24*^−/−^ mouse model has been described previously [22]. Primary MEFs were extracted from fetuses of 13.5-day-old pregnant female *Zmpste24*^+/−^ mice (C57BL background). Cells were cultured in DMEM/HIGH glucose medium containing 10% (vol/vol) fetal bovine serum (Gibco) and 1% penicillin/streptomycin incubated at 37 °C in a 5% CO_2_ humidified atmosphere.

### Drug preparation

All drugs (compounds in the DDR-related kinases inhibitor library- Custom compound library from Targetmol, USA.) were either prepared or provided as 10 mM stock solutions in DMSO. The compounds are list in Supplementary Table S1. They were then diluted in a culture medium to obtain a suitable working solution. Culture medium with DMSO at the same concentration was used as a negative control.

### Senotherapeutics asssay

MEFs (5 × 10^3^) at passage 6 were seeded in 96-well plates at least 6 h prior to treatment. After adding the drugs, the MEFs were incubated for 24–48 h. For fluorescence analysis of SA-β-Gal activity, cells were washed once with phosphate buffered saline, C_12_FDG (10 µM) was added to the culture medium, and the cells were incubated for 2 h. C_12_FDG fluorescence intensity was detected by Enspire 2300 Multimode Plate Reader (PerkinElmer, MA, USA). All samples were analyzed in duplicate with 3–5 fields per well and mean values and standard deviations were calculated accordingly.

### Cell viability assay

MEFs were seeded on 96-well plates for 24 h, and then incubated with test compounds for another 48 h at 37 °C and 5% CO_2_. Cell viability was assessed from the CellTiter 96® AQueous One Solution Cell Proliferation Assay (cat#G3581, Promega, WI, USA). Pipet 20 µl of CellTiter 96® AQueous One Solution Reagent into each well of the 96-well assay plate containing the samples in 100 µl of culture medium, incubating the plate at 37 °C for 2 h in a humidified, 5% CO_2_ atmosphere. Record the absorbance at 490 nm using Enspire 2300 Multimode Plate Reader (PerkinElmer, MA, USA).

### Plasmid construction and siRNAs

The full-length HP1α gene was subcloned into pcDNA3.1-3xFLAG vectors. CK2 was amplified from a cDNA library of HEK293 cells and cloned into pKH3-3xHA vectors. HP1α mutants were generated using a site-directed mutagenesis kit (New England Biolabs, USA). Transient transfection with these plasmids was performed using Lipofectamine 3000 (Invitrogen) according to the manufacturer’s instructions. Small interfering RNA (siRNA) against Ck2α or mock siRNA were synthesized by GenePharma (Shanghai, China). Transfection was performed using Lipofectamine RNAiMAX Transfection Reagent (Invitrogen) according to the manufacturer’s instructions. The primers used are listed in Supplementary Table S2.

### Western blot analysis

Total protein was isolated with modified RIPA buffer (0.1 M Tris-HCl, pH 7.5, 0.1 M NaCl, 1 mM EDTA, 1 mM DTT), phosphatase inhibitor, and protease inhibitor cocktail (Roche, Germany), quantified with a BCA protein assay kit (Invitrogen) and separated by SDS polyacrylamide gel electrophoresis followed by western blotting with the respective antibodies.

### Co-immunoprecipitation assay

For co-immunoprecipitation, cells that were subjected to the indicated treatments were lysed in lysis buffer (250 mM NaCl, 20 mM Tris-HCl pH 8.0, 2 mM EDTA, 10% glycerol, and 0.1% NP-40), supplemented with EDTA-free protease inhibitor (Roche). Whole-cell extracts were then incubated with the indicated antibody overnight at 4 °C. Bead-bound immunoprecipitates were washed with lysis buffer, boiled in a loading buffer, then analyzed by western blotting analysis.

### Flow cytometry

Senescent MEF cells from passage 6 were seeded at 70–80% confluence in 6-well culture plates and cultured for 24 h at 37 °C. For MEF, 5 × 10^5^ cells/well were used. Following the addition of the drugs, lysosomal alkalinization was induced by pretreating cells with 100 nM bafilomycin A1 for 1 h in a fresh cell culture medium at 37 °C. C_12_FDG (10 μM) solution was then added to the cell culture medium for 2 h. The cells were harvested by trypsinization and resuspended in 1× Annexin V buffer containing 5 µl of Annexin V-APC and 5 µl 7-AAD per 1 × 10^5^ cells/100 µl. The cells were analyzed by flow cytometry within 1 h. To estimate relative SA-β-Gal activity, a two-parameter display of forward scatter versus side scatter was set up, excluding subcellular debris. The events within this region were shown in a green fluorescence histogram, where the Y-axis indicates cell number and the X-axis indicates C_12_FDG fluorescence intensity on a log scale.

### Immunofluorescence staining

Cells were mounted on glass slides and fixed in 4% paraformaldehyde. The cells were then permeabilized with PBS containing 0.1% Triton X-100 for 15 min, and blocked with 1% bovine serum in PBS for 30 min at room temperature. The cells were then incubated with various primary antibodies overnight at 4 °C, and detected by Alexa Fluor conjugated secondary antibodies (Alexa −488, −594; 1: 500, Life Technologies) for 1 h at room temperature in the dark. The cells were then co-stained with 4’, 6-diamidino-2-phenylindole (DAPI) (1:10000 dilution) to visualize the nuclei. Images were captured under a confocal laser scanning microscope system (CarlZeiss). A representative image for each condition is shown.

### RNA isolation and quantitative RT-PCR

Total RNA was isolated using Trizol reagent (Invitrogen, 15596-026). RNA (2 μg) and reverse transcribed into cDNA using 5× Primescript RT Master Mix (Takara). Real-time quantitative polymerase chain reaction (RT-qPCR) was performed using 2× SYBR Green Mix (Takara) in a Bio-Rad detection system. Each sample was run in triplicate and gene expression levels were normalized to *Gapdh*. The primers are listed in Supplementary Table 2.

### TUNEL staining

TUNEL staining was assessed with a DeadEnd™ Fluorometric TUNEL System (G3250, Promega, WI, USA) according to the manufacturer’s instructions, and then images were captured under a confocal laser scanning microscope system (CarlZeiss).

### SA-β-Gal staining and apoptosis assays

SA-β-Gal staining was assessed with a cellular senescence assay kit according to the manufacturer’s instructions, and then photographed. Blue-stained MEFs were counted in more than 300 randomly chosen cells. Apoptosis was analyzed by flow cytometry as described previously [58].

### Animal studies

The *Zmpste24*^−/−^ mice used have been described elsewhere [22]. *Zmpste24*^+/+^ and *Zmpste24*^−/−^ mice were assigned to the vehicle or the TBB treatment groups, which were fed TBB mixed into their drinking water at a final concentration of 50 μM/L. Survival of TBB-treated or vehicle-treated *Zmpste24*^−/−^ mice and wild-type controls was recorded, and their body weight was monitored weekly. The survival curves of untreated and TBB-fed mice were compared by Kaplan–Meir analysis.

For bone density analysis, 4-month-old male mice were euthanized by decapitation. The thigh bone was fixed in 4% PFA at 4 °C overnight. The relevant data were collected by micro-CT (Scanco Medical, CT100). Each experimental group included at least three mice. All experiments using mice were performed in accordance with protocols approved by the Committee on the Use of Live Animals in Teaching and Research of Shenzhen University.

### Statistical analysis

All experiments were carried out with at least three replicates. All data values are expressed as mean ± standard deviation (SD) or mean ± standard error of the mean (SEM). For multiple comparison, one-way or two-way ANOVA were applied as indicated. Statistical analysis was done by Student’s *t* test, with a *p* < 0.05 considered statistically significant.

### Supplementary information

supplementary materials

## Data Availability

The data are available within the article, Supplementary Information or Original Data file.
