# Supplementary material for: Targeting CK2 eliminates senescent cells and prolongs lifespan in Zmpste24-deficient mice
Source: Cell Death Dis. 2024 May 30;15(5):380. doi: 10.1038/s41419-024-06760-0 (PMC11139886; doi:10.1038/s41419-024-06760-0)
Supplement: Supplementary file 1 — supplementary materials [file 41419_2024_6760_MOESM1_ESM.pdf]

A

	1	2	3	4	5	6	7	8	9	10	11	12
**a**	Empty	**321674-73-1**<br>BIBR 1532	**476-66-4**<br>Ellagic acid	**63585-09-1**<br>Foscarnet sodium	**93107-08-5**<br>Ciprofloxacin hydrochloride	**95058-81-4**<br>Gemcitabine	**522-51-0**<br>Dequalinium chloride	**119413-54-6**<br>Topotecan hydrochloride	**763113-22-0**<br>Olaparib (AZD2281, Ku-0059436)	**36791-04-5**<br>Ribavirin	**117-39-5**<br>Quercetin	Empty
**b**	Empty	**29767-20-2**<br>Teniposide	**86639-52-3**<br>SN-38	**587871-26-9**<br>KU55933	**61379-65-5**<br>Rifapentine	**39809-25-1**<br>Penciclovir	**404950-80-7**<br>Panobinostat (LBH589)	**1345675-02-6**<br>ETP46464	**912445-05-7**<br>ABT888 hydrochloride	**142217-69-4**<br>Entecavir	**1009820-21-6**<br>CX-4945 (Silmitasertib)	Empty
**c**	Empty	**344458-15-7**<br>PJ34 hydrochloride	**783355-60-2**<br>PCI-24781 (Abexinostat)	**17374-26-4**<br>TBB(NSC 231634)	**61281-37-6**<br>Schizandrin B	**52286-74-5**<br>Ginsenoside Rg2	**1012054-59-9**<br>CUDC-101	**325970-71-6**<br>L67	**209783-80-2**<br>Entinostat (MS-275)	**97682-44-5**<br>Irinotecan	**344458-19-1**<br>PJ34	Empty
**d**	Empty	**329907-28-0**<br>TTP 22	**209216-23-9**<br>Entecavir hydrate	**1232416-25-9**<br>VE822	**244767-67-7**<br>Dapivirine (TMC120)	**925701-49-1**<br>KU-60019	**1316214-52-4**<br>Rocilinostat (ACY-1215)	**912444-00-9**<br>Veliparib (ABT-888)	**154447-35-5**<br>NU7026	**301836-43-1**<br>D 4476	**95734-82-0**<br>Nedaplatin	Empty
**e**	Empty	**726169-73-9**<br>Mocetinostat (MGCD0103)	**186692-46-6**<br>Roscovitine (CYC202,Seliciclib)	**1232410-49-9**<br>VE-821	**252935-94-7**<br>CHIR-98014	**1095382-05-0**<br>CCT137690	**252917-06-9**<br>CHIR-99021	**112522-64-2**<br>CI994 (Tacedinaline)	**1223001-51-1**<br>Torin 2	**1403764-72-6**<br>PFI-1 (PF-6405761)	**1609960-30-6**<br>TH287 hydrochloride	Empty
**f**	Empty	**410536-97-9**<br>Sirtinol	**519-23-3**<br>Ellipticine	**537672-41-6**<br>UF010	**601514-19-6**<br>TWS119	**937039-45-7**<br>Pimelic diphenylamide 106	**84605-18-5**<br>Cycloastragenol	**75607-67-9**<br>Fludarabine Phosphate	**61276-17-3**<br>Verbascoside	**1352226-88-0**<br>Azd6738	**1219168-18-9**<br>Dorsomorphin dihydrochloride	Empty
**g**	Empty	**313516-66-4**<br>T0070907	**503468-95-9**<br>NU7441 (KU-57788)	**3544-24-9**<br>INO-1001	**1198097-97-0**<br>Mirin	**31083-55-3**<br>PRT 4165	**1190307-88-0**<br>Sofosbuvir (PSI-7977, GS-7977)	**4707-32-8**<br>Beta-Lapachone	**1169562-71-3**<br>XL413-hydrochloride	**125314-13-8**<br>CP21R7	**1604810-83-4**<br>THZ1	Empty
**h**	Empty	**161058-83-9**<br>NU2058	**327036-89-5**<br>TDZD-8	**869363-13-3**<br>MLN8054	**1054543-47-3**<br>Apoptozole	**891494-64-7**<br>SCH900776 S-isomer	**1228013-15-7**<br>CC-115	**168425-64-7**<br>Compound 401	**1796596-46-7**<br>NSC23005 Sodium	**924473-59-6**<br>T5601640	**1169558-38-6**<br>XL413	Empty

B

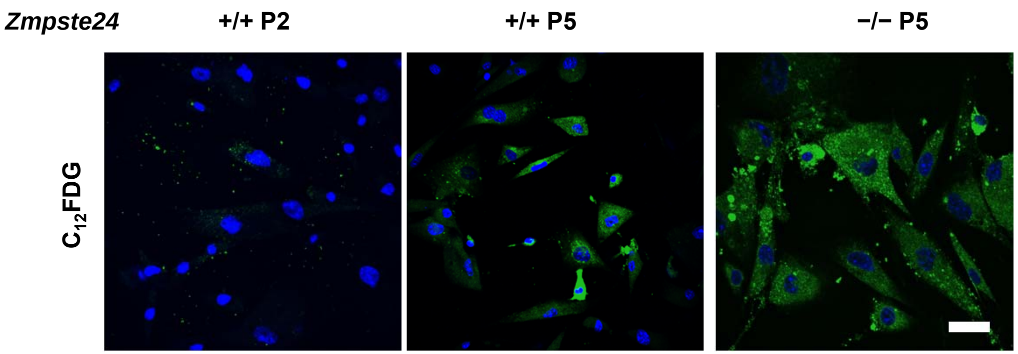

C

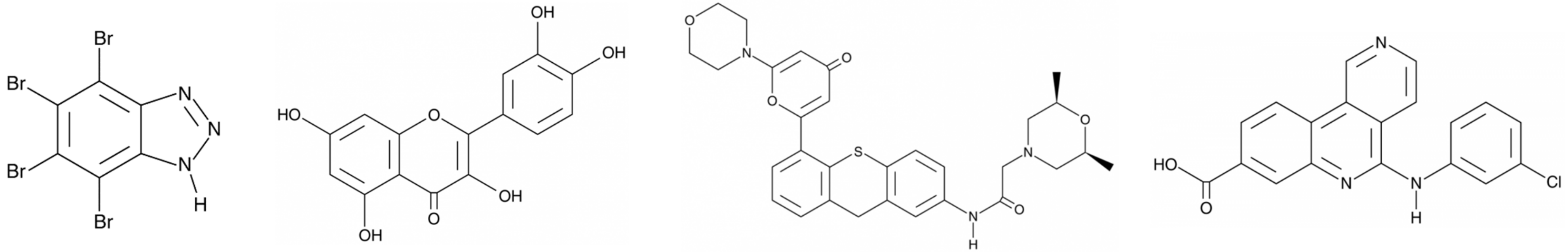

TBB **quercetin** **Ku-60019** **CX-4945**

Supplementary Figure 1. Classes of compound libraries for DDR-related kinase inhibitors
(A) Detail of the compound library of DDR-related kinase inhibitors. (B) Representative immunofluorescence confocal microscopy images from triplicate experiments of C_{12}FDG stained senescent cells. Scale bar, 10 μm. (C) Structure of 4,5,6,7-tetrabromo-2-azabenzamidazole (TBB), quercetin, Ku-60019 and CX-4945.

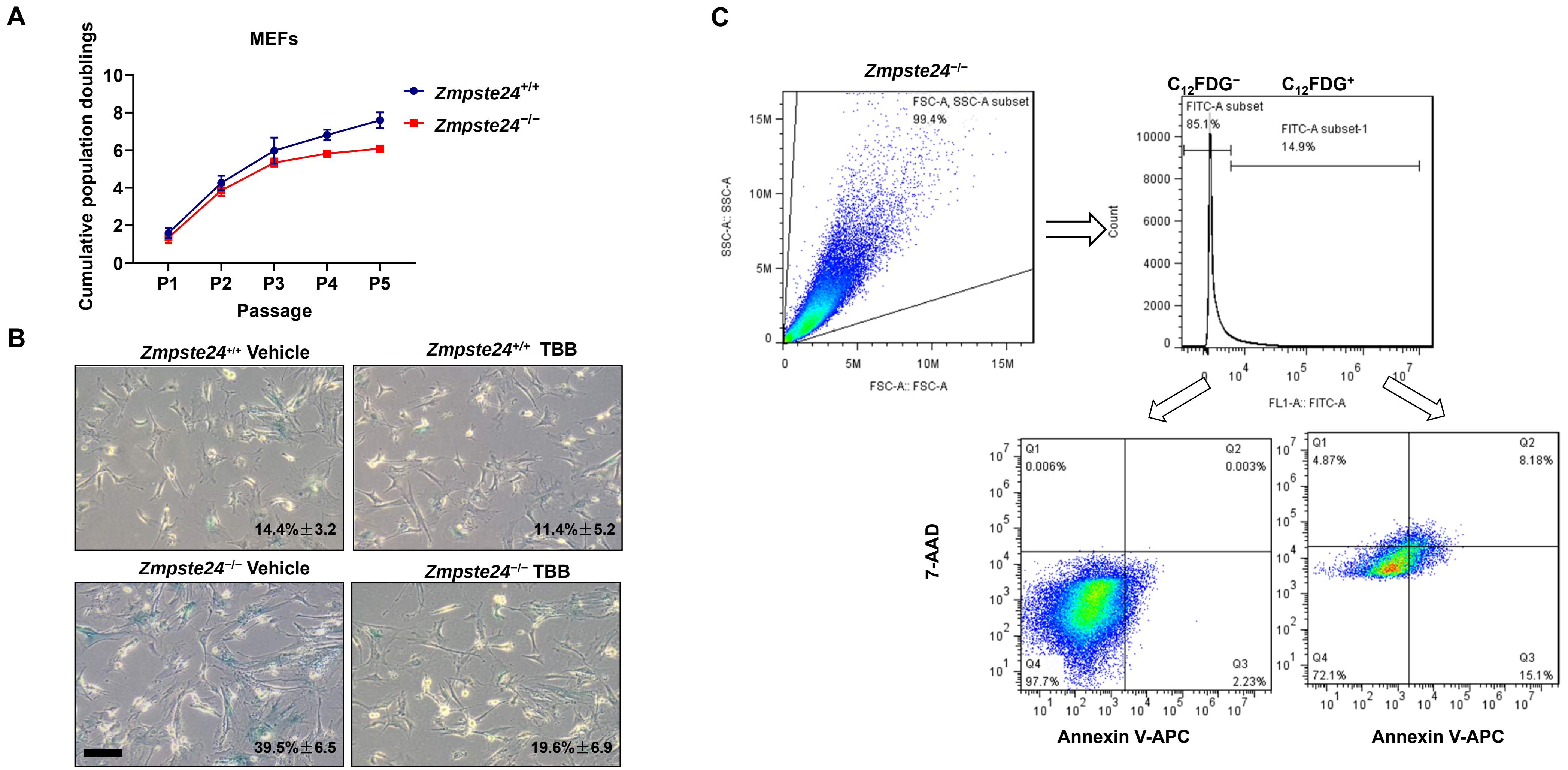

Supplementary Figure 2. TBB does not affect the apoptosis of nonsenescent cells

(A) Continuous passaging assay in MEFs showing *Zmpste24*$^{+/+}$ and *Zmpste24*$^{-/-}$ MEFs. (B) Representative images of SA-β-Gal activity showing blue-stained senescent cells in *Zmpste24*$^{-/-}$ MEFs and WT littermate controls treated with 75 μM TBB or DMSO (control) at passage 6. Scale bar, 100 μm. (C) Gating Strategy for C_{12}FDG-AnnexinV-7AAD Flow Cytometry. A two-parameter display of FSC versus SSC was set up to exclude subcellular debris. In a green fluorescence histogram C_{12}FDG fluorescence intensity was depicted in log scale. The percentage of positive cells was estimated by dividing the number of events within the bright fluorescence compartment by the total number of cells in the histogram. To estimate the number of live cells in SA-β-Gal positive and negative cells the subpopulation analyzed (C_{12}FDG positive cells or C_{12}FDG negative cells) was depicted on a two-parameter display of APC vs. PE-Cy5. The cells that were considered alive were those negative for APC (Annexin V-APC) and PE-Cy5 (7-AAD), AnnexinV-APC positive cells were considered apoptotic.

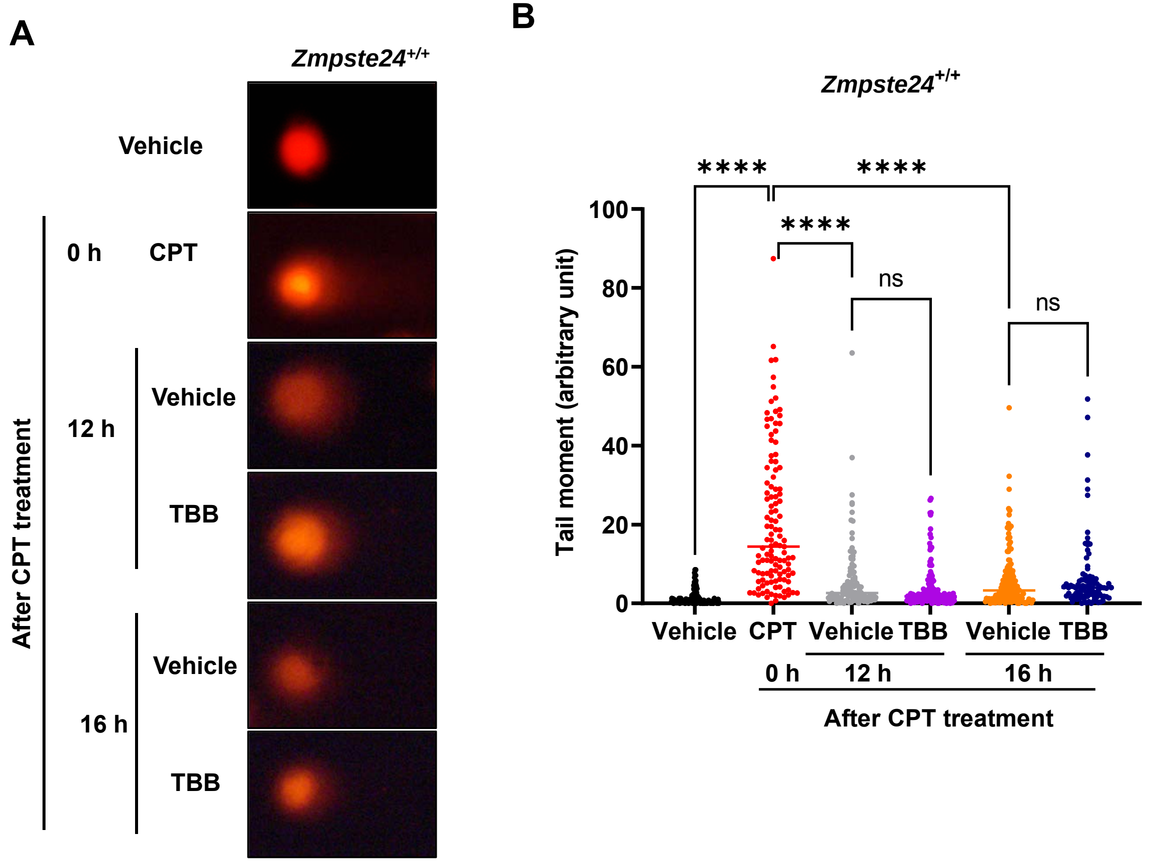

Supplementary Figure 3. TBB does not affect tail moment after CPT treatment in wild-type cells
Representative comet assay images at indicated time points after 4 μM camptothecin (CPT) for 1 h induced DNA damage or vehicle DMSO control in *Zmpste24*$^{+/+}$ MEFs pretreated with 75 μM TBB for 6 hours. (B) Tail moments quantified by Open Comet software after treatment with vehicle or TBB, as in A .

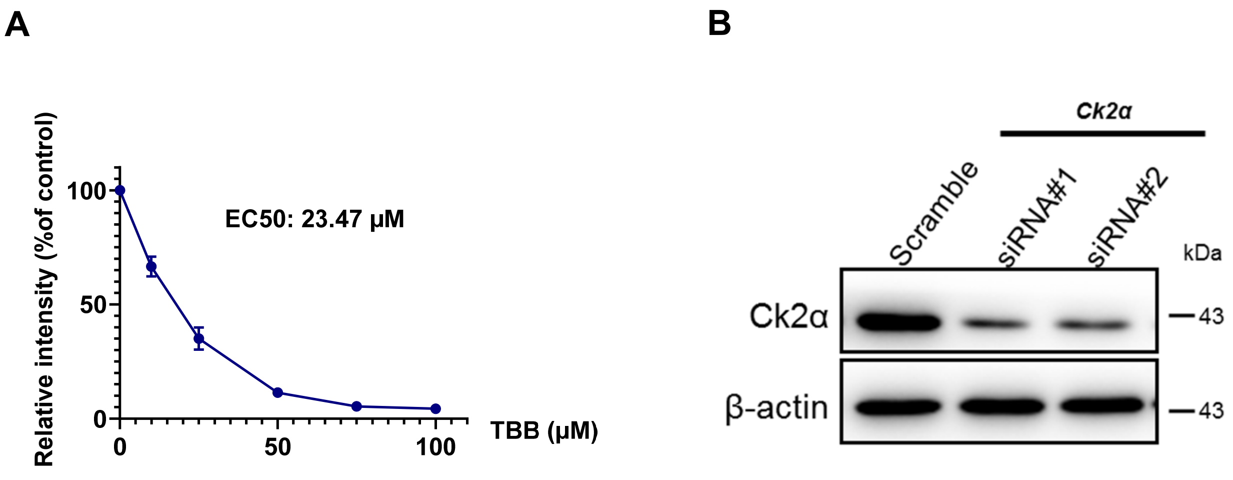

Supplementary Figure 4. TBB specifically inhibits the activity of CK2 *in vitro*
(A) Relative CK2 enzyme activity detection under 0–100 μM TBB treatment *in vitro*. Chart plots mean ± SD from independent triplicate experiments. (B) Representative immunoblots showing Ck2α and β-actin protein levels in wild-type MEF cells at passage 6 treated with si-CK2α or scramble siRNA.

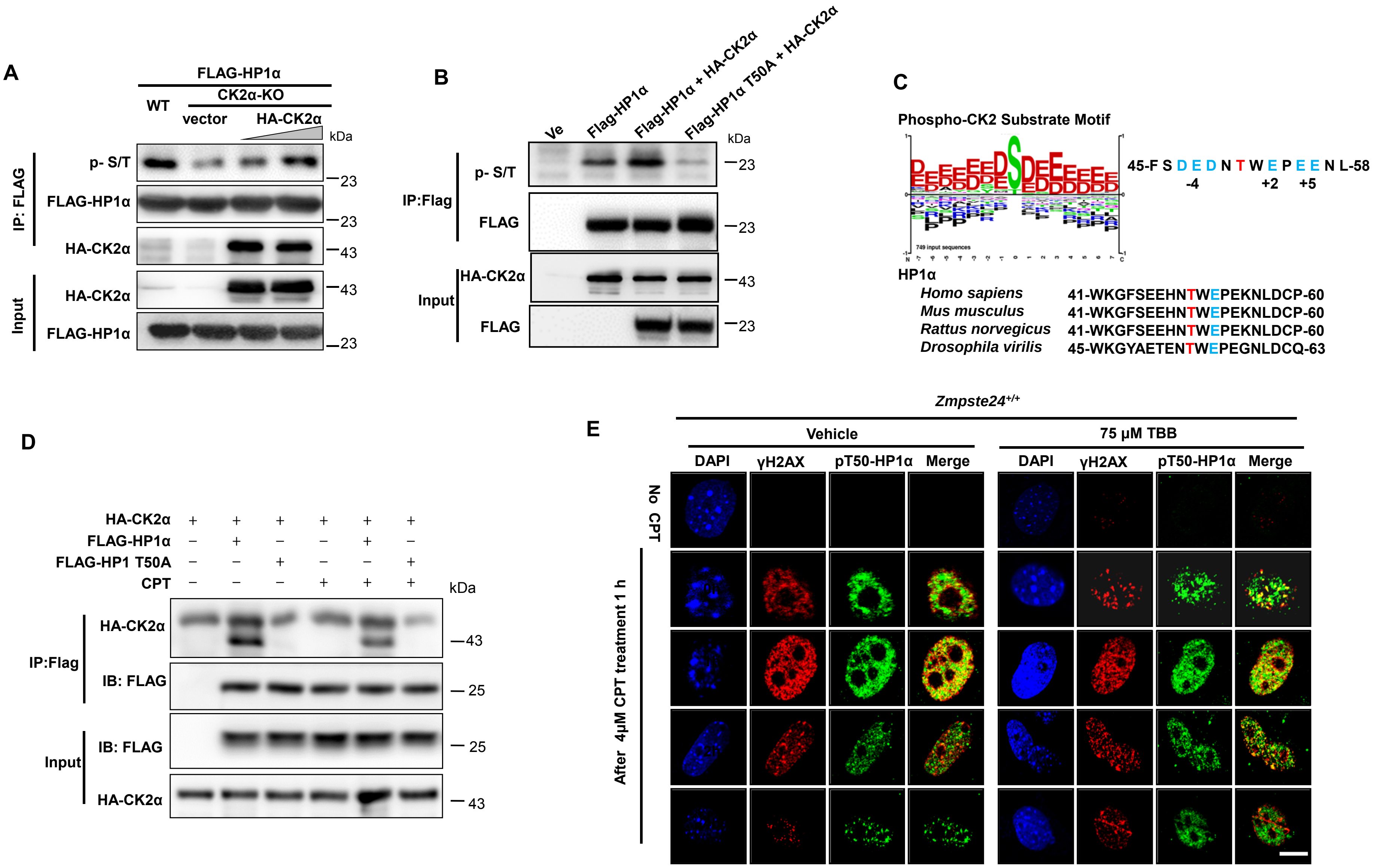

Supplementary Figure 5. CK2 phosphorylates HP1α at position T50

(A) Immunoblots showing p-S/T Q of FLAG-HP1α transfected HA-CK2α and FLAG-HP1α by using an anti-FLAG antibody in CK2α knockout HKE293 cells. The amount of CK2α plasmid was 0.1, 0.5, and 1 μg. (B) Immunoblots showing p-S/T Q of FLAG-HP1α or T50A transfected with HA-CK2α and FLAG-HP1α by using an anti-FLAG antibody in HKE293 cells. (C) Alignment of phosphorylation motifs of CK2 and HP1α at position T50 in different species. (D) Immunoblots showing pT50-HP1α and CK2α protein levels after treatment with 75 μM TBB or vehicle in CK2α knockout HKE293 cells and control cells. (E) Representative immunofluorescence confocal microscopy images of γ-H2AX and pT50-HP1α immunofoci recruitment in response to 4 μM CPT-induced DNA damage in TBB treated *Zmpste24*$^{+/+}$ MEFs. TBB inhibited the co-immunofoci of pT50-HP1α and γ-H2AX at the indicated time (after CPT treatment 0, 1, 2, 4 hours). Scale bar, 10 μm.

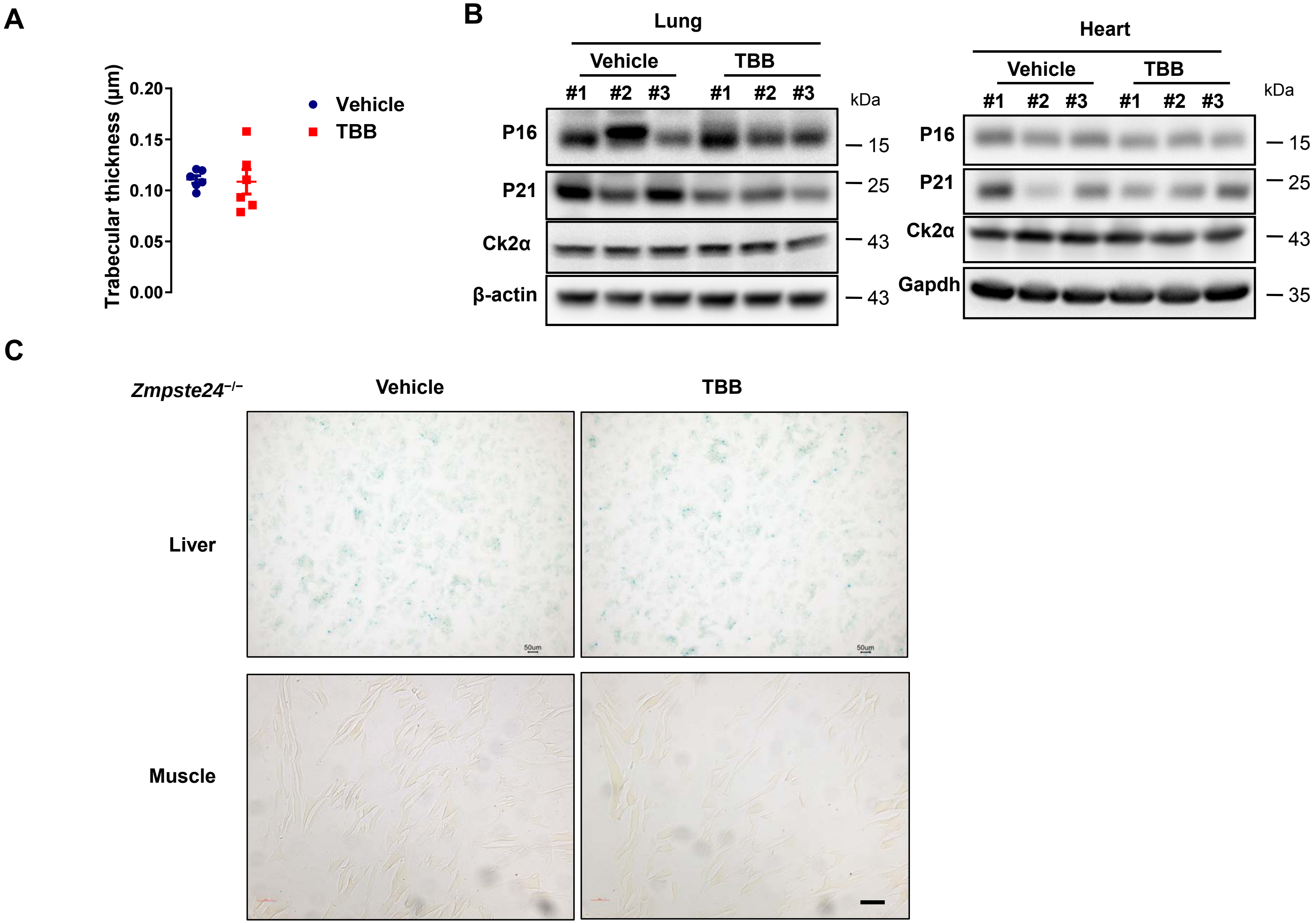

Supplementary Figure 6. TBB does not affect the thickness of trabecular bone in *Zmpste24*-deficient mice

(A) Micro-CT analysis showed no significant change in trabecular bone thickness in TBB-fed *Zmpste24*$^{-/-}$ mice. (B) Immunoblots showing P16 and P21 protein levels in lung and heart tissues isolated from male TBB-fed *Zmpste24*$^{-/-}$ and vehicle-fed control mice. (C) Representative photos of SA-β-gal activity showing blue-stained senescent cells of liver and muscle in TBB-fed *Zmpste24*$^{-/-}$ mice compared against those of vehicle-fed controls. Scale bar, 50 µm.

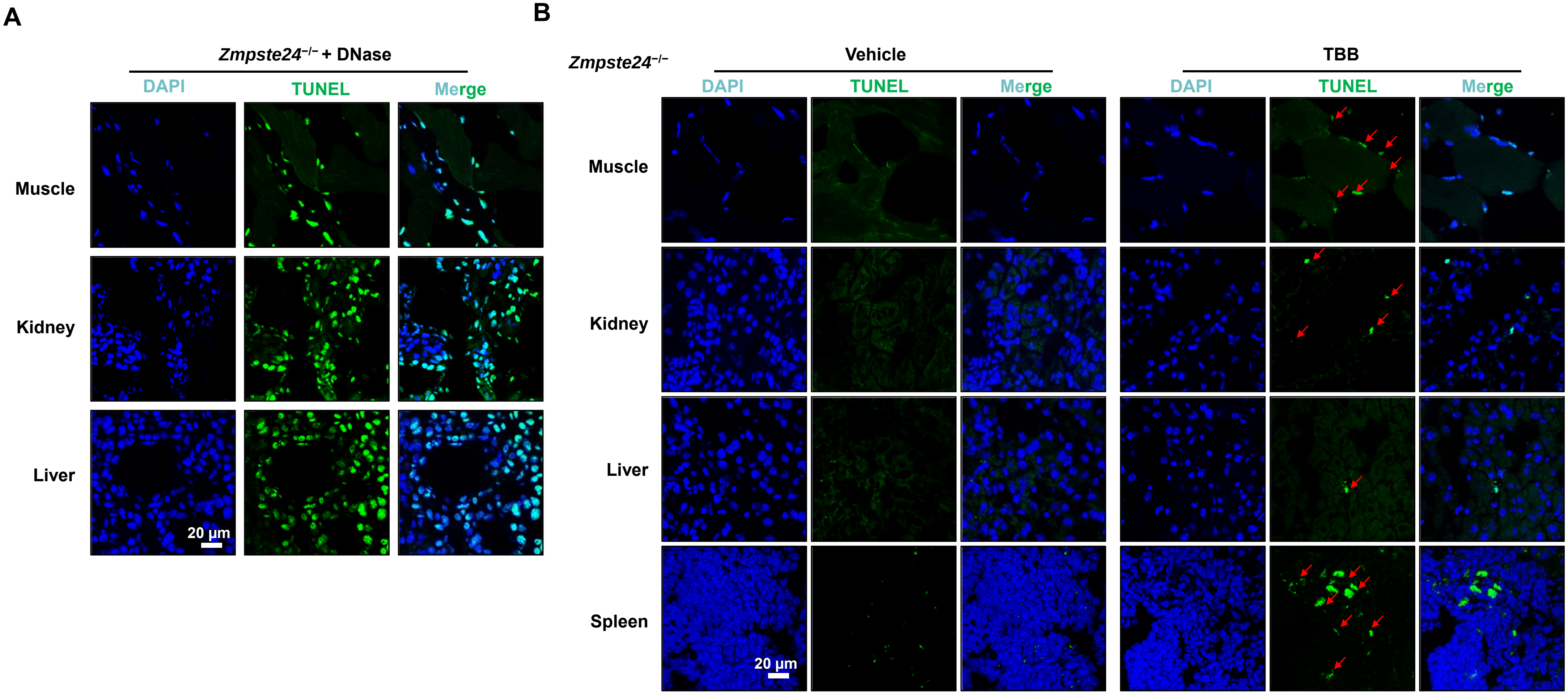

Supplementary Figure 7. (A) Frozen sections were stained for apoptosis in *Zmpste24*$^{-/-}$ mice, and DNase treatment was used as a positive control. (B) Frozen sections were used for tissue apoptosis staining in TBB-fed *Zmpste24*$^{-/-}$ mice were compared with vehicle controls, the red scissors indicated apoptotic signals. Scale bar, 20 μm.

Table S1. Compound List

Index	ID	MOLENAME	CAS	Formula	MolWt
1	T2380	BIBR 1532	321674-73-1	C21H17NO3	331.36
2	T0465	Ellagic acid	476-66-4	C14H6O8	302.19
3	T0220	Foscarnet sodium	63585-09-1	CNa3O5P	191.95
4	T0250	Ciprofloxacin hydrochloride	93107-08-5	C17H18FN3O3·HCl	367.8
5	T0251	Gemcitabine	95058-81-4	C9H11F2N3O4	263.2
6	T0979	Dequalinium chloride	522-51-0	C30H40Cl2N4	527.59
7	T1174	Topotecan hydrochloride	119413-54-6	C23H24ClN3O5	457.92
8	T3015	Olaparib (AZD2281, Ku-0059436)	763113-22-0	C24H23FN4O3	434.46
9	T0684	Ribavirin	36791-04-5	C8H12N4O5	244.2
10	T2174	Quercetin	117-39-5	C15H10O7	302.24
11	T1523	Teniposide	29767-20-2	C32H32O13S	656.66
12	T1703	SN-38	86639-52-3	C22H20N2O5	392.4
13	T2685	KU55933	587871-26-9	C21H17NO3S2	395.49
14	T1629	Rifapentine	61379-65-5	C47H64N4O12	877.03
15	T1643	Penciclovir	39809-25-1	C10H15N5O3	253.26
16	T2383	Panobinostat (LBH589)	404950-80-7	C21H23N3O2	349.43
17	T2084	ETP46464	1345675-02-6	C30H22N4O2	470.52
18	T2105	ABT888 hydrochloride	912445-05-7	C13H18Cl2N4O	317.21
19	T0085L	Entecavir	142217-69-4	C12H15N5O3	277.28
20	T2259	CX-4945 (Silmitasertib)	1009820-21-6	C19H12ClN3O2	349.77
21	T2124	PJ34 hydrochloride	344458-15-7	C17H18ClN3O2	311.8
22	T0431	PCI-24781 (Abexinostat)	783355-60-2	C21H23N3O5	397.42
23	T2695	TBB(NSC 231634)	17374-26-4	C6HBr4N3	434.71
24	T2925	Schizandrin B	61281-37-6	C23H28O6	400.46
25	T2873	Ginsenoside Rg2	52286-74-5	C42H72O13	785.01
26	T3108	CUDC-101	1012054-59-9	C24H26N4O4	434.49
27	T3103	L67	325970-71-6	C16H14Br2N4O4	486.11
28	T6233	Entinostat (MS-275)	209783-80-2	C21H20N4O3	376.41
29	T6228	Irinotecan	97682-44-5	C33H38N4O6	586.68
30	T6197	PJ34	344458-19-1	C17H17N3O2	295.34
31	T1828	TTP 22	329907-28-0	C16H14N2O2S2	330.42
32	T0085	Entecavir hydrate	209216-23-9	C12H15N5O3·H2O	295.3
33	T2669	VE822	1232416-25-9	C24H25N5O3S	463.55
34	T1815	Dapivirine (TMC120)	244767-67-7	C20H19N5	329.4
35	T2474	KU-60019	925701-49-1	C30H33N3O5S	547.67
36	T2489	Rocilinostat (ACY-1215)	1316214-52-4	C24H27N5O3	433.5
37	T2591	Veliparib (ABT-888)	912444-00-9	C13H16N4O	244.29
38	T2433	NU7026	154447-35-5	C17H15NO3	281.31
39	T2449	D 4476	301836-43-1	C23H18N4O3	398.41

40	T2410	Nedaplatin	95734-82-0	C2H8N2O3Pt	303.18
41	T2512	Mocetinostat (MGCD0103)	726169-73-9	C23H20N6O	396.44
42	T2095	Roscovitine (CYC202,Seliciclib)	186692-46-6	C19H26N6O	354.45
43	T3032	VE-821	1232410-49-9	C18H16N4O3S	368.41
44	T2608	CHIR-98014	252935-94-7	C20H17Cl2N9O2	486.31
45	T2611	CCT137690	1095382-05-0	C26H31BrN8O	551.48
46	T2310	CHIR-99021	252917-06-9	C22H18Cl2N8	465.34
47	T1888	CI994 (Tacedinaline)	112522-64-2	C15H15N3O2	269.3
48	T6100	Torin 2	1223001-51-1	C24H15F3N4O	432.4
49	T6222	PFI-1 (PF-6405761)	1403764-72-6	C16H17N3O4S	347.39
50	T2069	TH287 hydrochloride	1609960-30-6	C11H10Cl2N4·HCl	305.59
51	T6671	Sirtinol	410536-97-9	C26H22N2O2	394.47
52	T1166	Ellipticine	519-23-3	C17H14N2	246.31
53	T3205	UF010	537672-41-6	C11H15BrN2O	271.15
54	T2166	TWS119	601514-19-6	C18H14N4O2	318.33
55	T3193	Pimelic diphenylamide 106	937039-45-7	C20H25N3O2	339.43
56	T3333	Cycloastragenol	84605-18-5	C30H50O5	490.715
57	T6501	Fludarabine Phosphate	75607-67-9	C10H13FN5O7P	365.21
58	T3375	Verbascoside	61276-17-3	C29H36O15	624.59
59	T3338	Azd6738	1352226-88-0	C20H24N6O2S	412.51
60	T6146	Dorsomorphin dihydrochloride	1219168-18-9	C24H25N5O·2HCl	472.41
61	T6689	T0070907	313516-66-4	C12H8ClN3O3	277.66
62	T6276	NU7441 (KU-57788)	503468-95-9	C25H19NO3S	413.49
63	T6329	INO-1001	3544-24-9	C7H8N2O	136.15
64	T3134	Mirin	1198097-97-0	C10H8N2O2S	220.25
65	T3110	PRT 4165	31083-55-3	C15H9NO2	235.24
66	T6676	Sofosbuvir (PSI-7977, GS-7977)	1190307-88-0	C22H29FN3O9P	529.45
67	T6407	Beta-Lapachone	4707-32-8	C15H14O3	242.27
68	T6735	XL413-hydrochloride	1169562-71-3	C14H13Cl2N3O2	122.1
69	T3684	CP21R7	125314-13-8	C19H15N3O2	317.3
70	T3664	THZ1	1604810-83-4	C31H28ClN7O2	566.05
71	T3186	NU2058	161058-83-9	C12H17N5O	247.3
72	T6187	TDZD-8	327036-89-5	C10H10N2O2S	222.26
73	T6315	MLN8054	869363-13-3	C25H15ClF2N4O2	476.86
74	T3293	Apoptozole	1054543-47-3	C33H25F6N3O3	625.56
75	T3700	SCH900776 S-isomer	891494-64-7	C15H18BrN7	376.25
76	T3541	CC-115	1228013-15-7	C16H16N8O	336.35
77	T3586	Compound 401	168425-64-7	C16N15H3O2	281.31
78	T3589	NSC23005 Sodium	1796596-46-7	C13H16NNaO4S	305.324
79	T3960	T5601640	924473-59-6	C19H14F3N3O3	389.33
80	T3352	XL413	1169558-38-6	C14H12ClN3O2	289.06

Table S2. List of primers used in this study

Name	Sequence
**CK2α RNAi**	
si-m*Ck2α*-1 sense	5'-CGAGUUGCUUCUCGAUAUUU-3'
si-m*Ck2α*-1 antisense	5'-AAAUAUCGAGAAGCAACUCG-3'
si-m*Ck2α*-2 sense	5'-UGGACAAGCUGCUUCGAUAU-3'
si-m*Ck2α*-2 antisense	5'-AUAUCGAAGCAGCUUGUCCA-3'
**Protein expression**	
HP1α-flag-F	5'-GGTACCATGGGAAAGAAAACCAAGC-3'
HP1α-flag-R	5'-GCGTCTAGATTAGCTCTTTGCTGTTTC-3'
HP1αT50A-flag-F	5'-CAGGTTCCCAAGCATTGTGCTCCTCAGAAAAGCC-3'
HP1αT50A-flag-R	5'-GGCTTTTCTGAGGAGCACAATGCTTGGGAACCTG-3'
HP1αT50D-flag-F	5'-GTTTTTCTCAGGTTCCCAATCATTGTGCTCCTCAGAAAAGCCTTT-3'
HP1αT50D-flag-R	5'-AAAGGCTTTTCTGAGGAGCACAATGATTGGGAACCTGAGAAAAAC-3'
HP1α S11-14A-flag-F	5'-acatactcctcctcatcctctgcagcagcagcgtcagctgtccgcttggttttc-3'
HP1α S11-14A-flag-R	5'-gaaaaccaagcggacagctgacgctgctgctgcagaggatgaggaggagtatgt-3'
CK2α-HA-F	5'-AAGCTTATGTCGGGACCCGTGCCAA-3'
CK2α-HA-R	5'-TCTAGACTGCTGAGCGCCAGCGGCA-3'
**CK2α Knockout**	
sgCK2α-1-F	5'-CACCGCACACAGACCTCGAGAATAC-3'
sgCK2α-1-R	5'-AAACGTATTCTCGAGGTCTGTGTGC-3'
sgCK2α-2-F	5'-CACCGCTGGGATTACGAGTCACATG-3'
sgCK2α-2-R	5'-AAACCATGTGACTCGTAATCCCAGC-3'
**q PCR**	
mouse Gapdh-F(exon 2–3	5'-AATGGTGAAGGTCGGTGTG-3'
mouse Gapdh-R(exon 2–3	5'-GTGGAGTCATACTGGAACATGTAG-3'
mouse p16 (Cdkn2a) F	5'-CCCAACGCCCCGAACT-3'
mouse p16 (Cdkn2a) R	5'-GCAGAAGAGCTGCTACGTGAA-3'
mouse p21 (Cdkn1a) F	5'-GTCCAATCCTGGTGATGTCC-3'
mouse p21 (Cdkn1a) R	5'-GTTTTCGGCCCTGAGATGT-3'
mouse Ccl2-F	5'-ATTGGGATCATCTTGCTGGT-3'
mouse Ccl2-R	5'-CCTGGTGTTCACAGTTGCC-3'
mouse Ccl7-F	5'-CCTGGGAAGCTGTTATCTTCAAG-3'
mouse Ccl7-R	5'-CCTCCTCGACCCACTTCTGA-3'
mouse Ccl3-F	5'-CTCCCAGCCAGGTGTCATTTT-3'
mouse Ccl3-R	5'-CTTGGACCCAGGTCTCTTTGG -3'
mouse Cxcl5-F	5'-TGCCCTACGGTGGAAGTCATA-3'
mouse Cxcl5-R	5'-TGCATTCCGCTTAGCTTTCTTT-3'
mouse Il10-F	5'-ATAACTGCACCCACTTCCCA-3'
mouse Il10-R	5'-GGGCATCACTTCTACCAGGT-3'
mouse Cxcl10-F	5'-GCTGGGATTCACCTCAAGAA-3'
mouse Cxcl10-R	5'-CTTGGGGACACCTTTTAGCA-3'
